# Neural signatures of syntactic variation in speech planning

**DOI:** 10.1371/journal.pbio.3001038

**Published:** 2021-01-26

**Authors:** Sebastian Sauppe, Kamal K. Choudhary, Nathalie Giroud, Damián E. Blasi, Elisabeth Norcliffe, Shikha Bhattamishra, Mahima Gulati, Aitor Egurtzegi, Ina Bornkessel-Schlesewsky, Martin Meyer, Balthasar Bickel

**Affiliations:** 1 Department of Comparative Language Science, University of Zurich, Zurich, Switzerland; 2 Center for the Interdisciplinary Study of Language Evolution (ISLE), University of Zurich, Zurich, Switzerland; 3 Department of Humanities and Social Sciences, Indian Institute of Technology Ropar, Rupnagar, India; 4 Department of Computational Linguistics, University of Zurich, Zurich, Switzerland; 5 Human Evolutionary Biology Department, Harvard University, Cambridge, Massachusetts, United States of America; 6 Department of Linguistic and Cultural Evolution, Max Planck Institute for the Science of Human History, Jena, Germany; 7 Linguistic Convergence Laboratory, National Research University Higher School of Economics, Moscow, Russia; 8 Human Relations Area Files, Yale University, New Haven, Connecticut, United States of America; 9 Department of Psychology, University of York, York, United Kingdom of Great Britain and Northern Ireland; 10 School of Psychology, Social Work and Social Policy, University of South Australia, Adelaide, Australia; 11 Cognitive and Systems Neuroscience Research Hub, University of South Australia; 12 Division of Neuropsychology, Department of Psychology, University of Zurich, Zurich, Switzerland; 13 Cognitive Psychology Unit, Psychological Institute, University of Klagenfurt, Klagenfurt, Austria; New York University, UNITED STATES

## Abstract

Planning to speak is a challenge for the brain, and the challenge varies between and within languages. Yet, little is known about how neural processes react to these variable challenges beyond the planning of individual words. Here, we examine how fundamental differences in syntax shape the time course of sentence planning. Most languages treat alike (i.e., align with each other) the 2 uses of a word like “gardener” in “the gardener crouched” and in “the gardener planted trees.” A minority keeps these formally distinct by adding special marking in 1 case, and some languages display both aligned and nonaligned expressions. Exploiting such a contrast in Hindi, we used electroencephalography (EEG) and eye tracking to suggest that this difference is associated with distinct patterns of neural processing and gaze behavior during early planning stages, preceding phonological word form preparation. Planning sentences with aligned expressions induces larger synchronization in the theta frequency band, suggesting higher working memory engagement, and more visual attention to agents than planning nonaligned sentences, suggesting delayed commitment to the relational details of the event. Furthermore, plain, unmarked expressions are associated with larger desynchronization in the alpha band than expressions with special markers, suggesting more engagement in information processing to keep overlapping structures distinct during planning. Our findings contrast with the observation that the form of aligned expressions is simpler, and they suggest that the global preference for alignment is driven not by its neurophysiological effect on sentence planning but by other sources, possibly by aspects of production flexibility and fluency or by sentence comprehension. This challenges current theories on how production and comprehension may affect the evolution and distribution of syntactic variants in the world’s languages.

## Abstract 2

Little is known about the neural processes involved in planning to speak. This study uses eye-tracking and EEG to show that speakers prepare sentence structures in different ways and rely on alpha and theta oscillations differently when planning sentences with and without agent case marking, challenging theories on how production and comprehension affect language evolution.

## Introduction

Language is not a disparate and haphazard collection of idiosyncratic templates for how to formulate sentences. Instead, sentence templates form intricate systems of partial overlaps and alignments, an observation that has fueled inquiry since the Indian scholar Pāṇini wrote the first explicit grammar of a language over 2,500 years ago. For example, sentences like “The gardener planted a tree,” “The gardener crouched,” “The gardener worked hard,” or “A tree was planted by the gardener” align with each other in an abstract way by employing templates that begin with the same structure, here, with a noun phrase (the subject: “the gardener” and “a tree”) followed by a verb (“plants,” “crouches,” “works,” and “was”), while they differ in the remainder. Such alignments differ between languages in striking ways.

**Fig 1:**
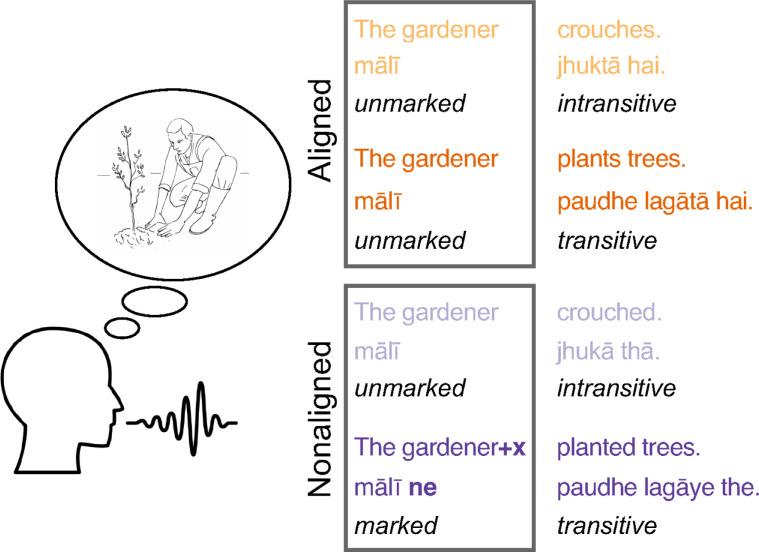
Illustration of the contrast between aligned and nonaligned agent expressions in Hindi, which allows both types of expressions (for detailed examples see S1 Table). In aligned expressions (used with imperfective aspect), unmarked noun phrases are compatible with both intransitive (“crouches”) and transitive sentences (“plants trees”). In nonaligned expressions (used with perfective aspect), unmarked noun phrases are only compatible with intransitive sentences (“crouched”) because in transitive sentences (“planted trees”) noun phrases are marked by an additional element “*+x*” (“ne” in Hindi).

Some languages align noun phrases like “the gardener” in a sentence with 1 noun phrase (intransitives, e.g., “the gardener crouched”) with the agent noun phrase in sentences with 2 or more noun phrases (transitives, e.g., “the gardener planted trees,” “the gardener poured water into the trough,” etc.). Other languages do not align the 2 sentence types and instead add a special marker in sentences with more than 1 noun phrase, keeping them formally distinct from sentences with only 1 noun phrase. These languages contrast a plain, unmarked noun phrase in intransitives (“the gardener crouched”) with a marked agent noun phrase in transitives (“the gardener*+x* planted trees,” with an additional marker represented here by “*+x*”; cf. Fig 1). This phenomenon, known as ergativity [1,2], is found in about one-third of the world’s languages [3].

Alignments and other kinds of partial overlaps are recognized as central properties of the human language faculty in all theories of grammar, albeit using widely different representational formats, e.g., derivations or inheritance schemata [4]. Despite their prominence in linguistic theory and the striking nature of variation, however, alignments have received remarkably little attention in neuroscience and psychology. The difference between aligned and nonaligned expressions poses 2 unresolved questions for neural processing and its relation to the variation between languages:

First, are there differences in the neural planning processes for aligned and nonaligned expressions? More concretely, is the way speakers plan sentences shaped by whether initial noun phrases are unmarked (“the gardener…,” aligned expression) or whether there is an opposition between unmarked and marked noun phrases (“the gardener” versus “the gardener*+x*,” nonaligned expression; Fig 1)? Does this difference affect sentence planning already in its early phases or only later, when speakers encode the phonological form of and articulatory motor plans for words with and without an additional agent noun phrase marker “*+x*”) [5,6]? Specifically, sentence structures with aligned expressions might allow speakers to delay commitment to the choice between an intransitive and a transitive sentence plan because their beginnings overlap (“the gardener… crouches/plants trees”) [7,8]. At the same time, this intermittent compatibility with multiple sentence plans might also require that 2 possible options need to be kept distinct while speakers construct a syntactic plan [9,10].

Second, if neural information processing is sensitive to how structures are aligned, does the neural processing mirror the observation that aligned structures are structurally simpler (with no additional marking) and more common in the world’s languages [11]? Such a mirroring has been motivated by evidence from event-related potentials in sentence comprehension: Across languages, both with and without nonaligned expressions, comprehenders initially interpret unmarked initial noun phrases as referring to agents, i.e., following an aligned pattern [3,12–16]. A bias for aligned structures is also found in phylogenetic models of linguistic evolution [3]: After controlling for contingencies of history (such as language contact and language shifts), languages are universally more likely to develop and maintain aligned expressions than the opposite. Such a correlation of neural processing and evolutionary developments might stem from a tight interlacing of comprehension and production processes [17] or from shared neural underpinnings [18,19], and would be consistent with other findings on how the brain’s processing constraints shape the form of languages [20–24].

Here, we seek to resolve these questions by exploiting a contrast between aligned and nonaligned syntax in Hindi for an experiment on sentence planning. Hindi aligns noun phrases in what is known as the imperfective aspect system (“the gardener crouches/was crouching” and “the gardener plants/was planting trees”) and keeps them nonaligned in the perfective aspect system (“the gardener crouched/has crouched” versus “the gardener*+x* planted/has planted trees,” Fig 1).

Speakers of Hindi described pictures of 1-participant (intransitive) and 2-participant (transitive) events by using sentences with either aligned or nonaligned syntax, with alignment condition split between groups of participants. While they prepared their responses, i.e., planned their sentences, we measured neural oscillatory processing using electroencephalography (EEG) simultaneously with overt visual attention as reflected in eye gaze. Visual attention allocation during planning is related to the syntactic structures being planned [25–28] and thus indicates how contrasts in structures shape the preparation of speakers’ sentence plans. With respect to neural processes, we focus on changes in total oscillatory power (event-related desynchronization [ERD] and event-related synchronization [ERS], [29]) in the theta and alpha frequency bands, typically ranging from 3 to 7 Hz and 8 to 12 Hz, respectively. While subserving a wide range of processes [30], these frequency bands are implicated in the processing of syntactic (and possibly semantic) dependencies during the comprehension of sentences (theta: [31–39]; alpha: [34,35,40–42]). ERD and ERS are currently among the most suitable means to study the neural processes underlying early stages of sentence planning because oscillatory power can capture both evoked (phase-locked) and induced (not phase-locked) responses [43,44]. This allows the effective study of neural events involved in sentence planning that either begin at the moment when the to-be-described picture is presented or emerge during the process of sentence planning (and are therefore not phase-locked).

We target relational and structural planning processes in the first 800 ms after the picture onset, in line with crosslinguistic results on eye tracking during sentence planning [45,46]. Under the hypothesis that different processes underlie the preparation of aligned and nonaligned structures, we expected to find behavioral and neural dissociations in these early stages of planning. Specifically, we expected differences between sentence types in gazes toward agents as well as in neural oscillatory activity in the theta and alpha bands. These effects are taken to reflect speakers’ differential engagement of working memory and attentional processes [36,47–51] when committing to a specific structure earlier or later during the planning of aligned and nonaligned sentences.

## Results

**Fig 2:**
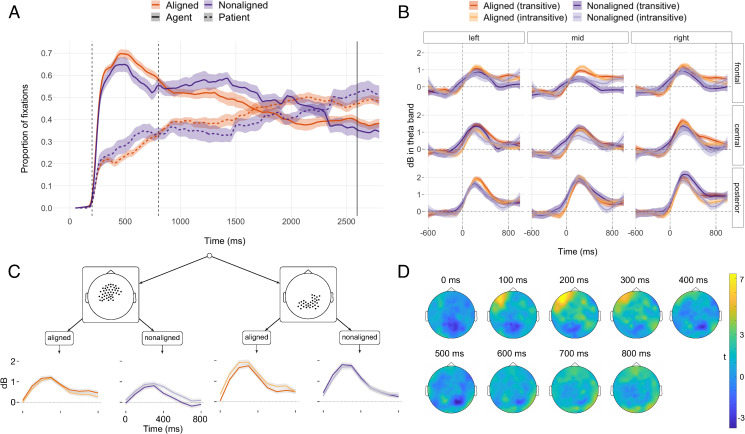
(A) Smoothed grand average of proportions of eye fixations to agent and patient characters in the picture stimuli during planning of transitive sentences (“The gardener plants trees” and “The gardener*+x* planted trees”). The solid vertical line represents speech onset (grand mean of transitive sentence responses). Overall, the planning of aligned sentences reveals more visual attention to agents (*p* < 0.001 in linear mixed-effects regression). (B) Smoothed grand average of event-related power changes (dB relative to a baseline period of −600 to −200 ms) in individually defined theta frequency bands. Grid cells in this panel represent ROIs (see S7 Table for details). Dashed vertical lines (in A and B) indicate analysis time windows, ribbons indicate standard errors, and ***t* = 0** is the onset of stimulus picture presentation. (C) Regression model tree for power changes in individually defined theta frequency bands between 0 and 800 ms. Scalp maps show electrode positions of ROIs included in the respective grouping. All splits are statistically significant at *p* < 0.001. Tree tips show model fits for the respective grouping; ribbons represent 95% CIs, and colors code the same distinctions as in A and B. The model also identified lower-order splits which are subsidiary to the main difference between aligned and nonaligned sentences (see S4 Fig, S5 Table). (D) Topographic maps of power differences of aligned minus nonaligned sentences in *t*-values for a fixed theta frequency band (3–7 Hz). (Underlying data and scripts are available from https://osf.io/uhtcn/ and in the Supporting information file S1 Data.) Overall, the planning of aligned sentences reveals ERS (more positive dB) in the theta band. ERS, event-related synchronization; ROI, region of interest.

In the first 800 ms after picture onset, speakers fixated on the agent characters in the pictures more when planning aligned than when planning nonaligned sentences (Fig 2A). This is reflected in an interaction between the cubic time term and sentence type in a logistic mixed-effects growth curve regression [52] ($\hat{\beta }$ = 1.13, SE = 0.26, 95% CI = [0.63, 1.63], *p* < 0.001, S4 Table). EEG oscillatory power changes were analyzed with mixed-effects regression trees [53], which partition the data set by sentence type and regions of interest (ROIs; averaging across electrode positions within these regions) with respect to the time course of power changes, analyzed as polynomial growth curves (see Materials and methods). Our model identifies broadly distributed ERS in the theta band (Fig 2 B–D, S5 Table). Both intransitive and transitive aligned sentences exhibited stronger theta ERS than nonaligned sentences, starting from around 200 ms, especially over mid-frontal electrode sites, as well as subsisting after 600 ms in right-central and posterior electrode sites (Fig 2C and 2D).

**Fig 3:**
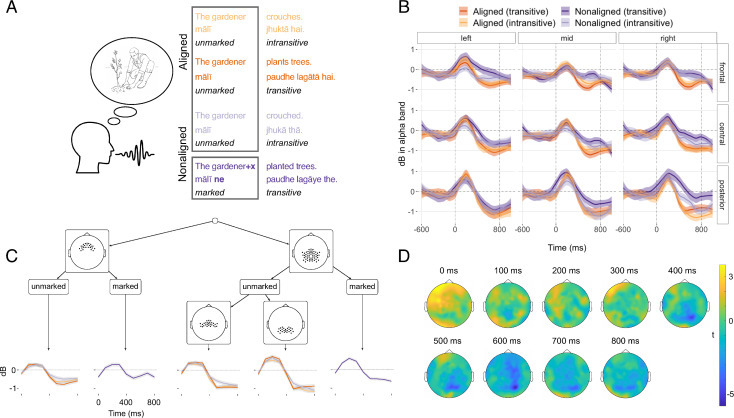
(A) Illustration of the contrast between unmarked and marked noun phrases (which crosses over aligned and nonaligned expressions) in Hindi: Aligned expressions are always unmarked (“The gardener…”), as are nonaligned noun phrases in intransitive sentences (“The gardener crouched”), while nonaligned noun phrases in transitive sentences are marked (“The gardener+x planted trees”). (B) Smoothed grand averages of event-related power changes (dB relative to a baseline period of −600 to −200 ms) in individually defined alpha frequency bands. (C) Regression model tree for power changes in individually defined alpha frequency bands between 0 and 800 ms. The model also identified lower-order splits which are subsidiary to the main difference between marked and unmarked expression (see S5 Fig, S6 Table). (D) Topographic maps of power differences (in *t*-values) between sentences with marked unmarked expressions for a fixed alpha frequency band (8–12 Hz). (A–D) All other conventions are identical to the conventions in Fig 2. (Underlying data and scripts are available from https://osf.io/uhtcn/ and in the Supporting information file S1 Data.) Overall, the planning of sentences with unmarked expression (intransitive and transitive aligned sentences and intransitive nonaligned sentences) reveals ERD (more negative dB) in the alpha band. ERD, event-related desynchronization.

In the alpha frequency band, we found a related but different effect (Fig 3, S6 Table). Here, the main distinction is between all marked and all unmarked noun phrases (Fig 3A), rather than between aligned and nonaligned syntax (Fig 1). Specifically, we found broadly distributed ERD starting after around 400 ms and peaking around 700 ms (Fig 3B), which was larger in sentences with unmarked noun phrases (“The gardener…”) than in sentences with marked noun phrases (“The gardener*+x*…”). The alpha ERD effect was most pronounced over central and posterior regions (Fig 3C and 3D).

All models controlled for a series of potential confounds and group-level variables (see Materials and methods). We specifically also controlled for (cumulative) syntactic priming, the phenomenon that the repetition of sentence structures alters and eases their subsequent processing [54,55]. We included the position of each utterance in the experiment as predictor so that effects persisted above and beyond any priming effects that may accumulate over an experimental session.

There were no differences in reaction times, i.e., speech onset latencies, between the production of aligned and nonaligned sentences (see S2 Fig, S3 Table). This suggests that the task demands were indistinguishable between describing the same picture as representing an ongoing (imperfective) or a recently completed (perfective) situation. Even though we cannot rule it out categorically, the eye tracking and EEG results are therefore unlikely to stem from the fact that the planning of aligned sentences necessitated a completed conceptualization of the depicted events, while the planning of nonaligned sentences necessitated an ongoing conceptualization of the same event.

Our choice of the analysis time window makes it unlikely that we measured the encoding of phonological word forms rather than differences in syntax. It is generally assumed that the generation of grammatical structure precedes lexical encoding and the planning of phonological word forms [26,56,57], although the exact timing of the switch from grammar to phonology is not well understood [58,59]. In line with this, phonological encoding is unlikely to have influenced grammatical encoding in our measurements because variation in phonological lengths of noun phrases (in syllables) has only small effects in our models, magnitudes smaller than the effects of the sentence type differences (S4–S6 Tables). Furthermore, our analysis time window and frequency bands largely guard against possible confounds from the articulatory encoding of phonological forms in terms of speech-related muscle movement plans since these processes set in only close to actual speech onset [57,60], which occurred at least 700 ms after our analysis window (see Materials and methods), and dominantly affect frequencies that are higher (>20 Hz [61]) than the alpha and theta bands we target. Finally, in single word production, theta band ERS and alpha band ERD specifically index the retrieval of conceptual and lexical information from memory [60,62] also when the phonological form of words is held constant.

## Discussion

Our results suggest that the planning of different types of sentences is neurally implemented in distinct ways. This is reflected by differences in the temporal dynamics of visual attention allocation and neural oscillatory power changes in the theta and alpha frequency bands. We propose that the observed pattern of results stems from 2 separate processes.

First, speakers directed more visual attention to agents and exhibited greater theta ERS when planning aligned than when planning nonaligned sentences (Fig 2). This suggests that they used different planning strategies for the preparation of aligned and nonaligned sentences. Speakers need to commit to a sentence plan earlier in the nonaligned condition because intransitive and transitive sentences already differ in the expression of the first noun phrase (with or without the additional agent marker “ne”). This requires speakers to prioritize early relational (and possibly structural) planning processes to encode who does what to whom [25,26]. This early (structural-)relational planning is reflected in looks that are more distributed over the whole picture and thus less focused on agents. In aligned sentences, by contrast, speakers do not need to decide on the full sentence plan as early [7]. The formal overlap between intransitive and transitive agent expressions allows speakers to delay their decision for 1 structure or the other. Speakers were thus able to primarily fixate on the agent as the action initiator [63] and as the referent of the first noun phrase in the sentence. In this way, speakers delayed the completion of relational processing [46] and held available all (aligned) structures that were compatible with a sentence starting with an unmarked agent noun phrase [10]. This scenario yields a natural interpretation of the increased theta ERS in the aligned condition as reflecting the simultaneous and noncommittal engagement of multiple compatible structures.

Intriguingly, sentence comprehension research has revealed that apart from being modulated by semantic and syntactic processing demands [32,33,37,38,64], theta oscillations play a role in maintaining working memory representations [34–37] and are linked to information retrieval from working memory [50,65]. Specifically, theta ERS is sensitive to the number of alternatives to be retrieved [50,66,67], in line with our proposed scenario of how aligned sentences are planned. The timing and frontal–parietal topography of theta ERS in our data also matches with frontal midline theta effects observed during the retrieval of syntactic structures in sentence comprehension [36].

The difference we detect in speakers’ commitment to sentence plans is consistent with behavioral findings on the importance of syntactic dependencies for the time course of planning: Like in the Hindi aligned condition, speakers of English and Japanese (where agent noun phrases are always aligned) plan agent noun phrases without an initial commitment to a verb and a sentence structure [68–70]. This contrasts with the earlier commitment that is required by sentences where the first noun phrase opens a strong dependency with the remaining sentence. In Hindi, we found this effect with marked, nonaligned noun phrases, and it is paralleled by the early commitment speakers need to make when planning patient noun phrases in English and Japanese which structurally depend on the verb [68–70]. More generally, the contrast we find between structures in Hindi expands previous findings that different dependencies afford different patterns in sentence planning [25,27,45,46,71–74].

Second, the planning of sentences with unmarked noun phrases induced larger ERD in the alpha band (Fig 3). We interpret this effect to reflect speakers’ need to keep distinct structures that share the same form (namely an unmarked noun phrase) at the beginning of sentences. This can be achieved by increased active neural information processing [29,75,76], e.g., in the form of increased engagement of cortical networks that are involved in processing syntactic information [40,42]. Converging evidence for our interpretation of the alpha ERD effect comes from reaction time findings that suggest higher processing loads when speakers need to separate alternative plans during sentence planning [9,10]. The need to keep distinct overlapping unmarked structures might also have contributed to the increased fixations to unmarked agents (Fig 2A) as longer fixation durations can indicate increased processing [49,77].

Our interpretation of the alpha ERD effect as reflecting the processing of syntactic alternatives is further supported by its central–posterior topography and its latency, with the largest differences setting in around 600 ms. The topography and latency are similar to alpha ERD effects associated with syntactic processes in sentence comprehension [34,35,40–42]. The current topographies are at the same time consistent with neuromodulatory evidence on the role of theta ERS and alpha ERD in controlling working memory–related processes [47]. Moreover, in conversation, alpha ERD is associated with shifting from comprehending interlocutors’ turns to preparing one’s own production [78,79]. Our results show that the observed alpha ERD effects go beyond mere attention shifts to production and are also sensitive to differences in syntactic planning processes between sentence types.

Our finding that theta power increases are associated with alpha power decreases fits with previous reports on memory processes outside language [80]. However, our results also suggest a small timing difference: While alpha ERD was most pronounced toward the end of our analysis time window, around 700 ms, theta ERS was most pronounced around 300 ms. This could reflect that speakers decided on which structure to produce in aligned contexts probably between 300 and 700 ms after the start of sentence planning. Thus, while eye tracking allows the detection of distinct steps in the planning process, our concurrent multimodal approach demonstrates that the time course of sentence planning can be characterized in greater detail with additional evidence from highly time-resolved electrophysiological recordings. The combination of eye gaze and neural oscillatory power changes opens up the possibility of additional insights into the fine-grained structure of sentence planning in future studies.

The origin of oscillatory activity can be exogenous or endogenous for the comprehension and perception of language [81–83]. For language planning and production, however, there is no exogenous source that would obviously give rise to power changes in the theta and alpha bands. Participants were “externally stimulated” by pictures that were to be described—the same ones for aligned and nonaligned sentences. Theta ERS and alpha ERD in the planning of different sentence types therefore most likely reflect the internal processing of sentence structures, building on memory-related control processes [62].

The increased neural activity in the planning of aligned and unmarked syntax (in terms of theta ERS and alpha ERD) contrasts with the apparent simplicity and frequency of systems with aligned expressions among the world’s languages. This challenges theories that propose sentence production processes as the key drivers of language form and the distribution of variants in language use [84,85]. These theories are based on the observation that speakers shape language use through choosing sentence structures that cause the least production difficulty, specifically, by placing easier to retrieve words first [86–88] and by reducing interference from similar words and structures [84,89,90]. Other structures would then be less likely to be produced and could only be used in restricted contexts or disappear from a language over time [84].

Our findings suggest either that (i) the relevant notion of production ease is independent of the neural activity we found and is instead grounded in more global constraints on sentence planning or that (ii) the distribution of aligned expressions in the world’s languages is not driven by sentence planning processes.

Under the first possibility, what matters most for speakers is fluency in sentence planning. Delaying the commitment to 1 grammatical structure is arguably beneficial for speakers’ fluency as it allows more flexibility when creating and implementing sentence plans incrementally [88,91]. Aligned expression facilitates this. The resulting benefit, e.g., for rapid response planning in conversation [78,92,93], would then be more important for language evolution than the increase in neural activity we found under aligned expression and the interference effects that this might generate.

The second possibility derives the prevalence for aligned expression from comprehension, specifically from a general preference of the sentence comprehension system to expect sentences to start with an unmarked noun phrase in the agent role [12–16]. Intriguingly, this expectation extends to languages with nonaligned syntax. For example, the nonaligned syntax of Hindi leads to unmarked noun phrases that regularly denote patients instead of agents in transitive sentences (e.g., the patient “paudhe” “trees” in Fig 1 is unmarked). When these noun phrases occur initially in a sentence (as they often do), the comprehension parser nevertheless assigns them an agent role transiently and then shows an electrophysiologically detectable effect of reanalysis and prediction failure at the position of the verb [3]. This incurs an additional processing demand [94,95] that might explain the bias against nonaligned syntax in the world’s languages.

Under this view, differences in planning processes between types of sentences have much less of an effect on linguistic distributions than processes during sentence comprehension. This is in line with theories of sound change that locate such effects chiefly in perception rather than production [96]. Prediction failure and reanalysis effects in comprehension would thus exert a vastly greater pressure on languages to abandon these sentence structures over time than the increased processing demands during the planning of aligned structures. This view is consistent with proposals that processing differences related to working memory engagement are key constraints of language evolution [20,21,97,98], to the extent that these differences stem from comprehension.

Alternatively, comprehension and production might form a trade-off: While the comprehension system favors unmarked, aligned syntax, production favors marked, nonaligned syntax, although to a much weaker extent. This would explain why nonaligned systems exist at all, despite their lower probability to emerge and persist during the evolution of languages [3].

More research is now needed to resolve these questions and to probe contrasts in alignment across diverse syntactic structures and languages. Our results demonstrate how new insights on the relationship between language production and the simplicity and prevalence of specific language structures critically rely on evidence from neural processes that underlie different syntactic variants in the earliest stages of sentence planning. This opens new avenues for research on the neural processes in language planning and speech production that go beyond the level of individual words [62,99,100] and beyond the small set of languages that have dominated the field so far [22,71,74,101–103].

## Materials and methods

### Participants

Fifty healthy, right-handed students at the Indian Institute of Technology Ropar with normal or corrected-to-normal vision (6 female, mean age = 20.47 years, SD = 3.35 years) participated in the experiment for payment and gave written informed consent. All participants were native speakers of Hindi, grew up in Uttar Pradesh, Madhya Pradesh, or Delhi, and reported to speak or grew up speaking Hindi to their parents on a daily basis. The study was approved by the Institutional Ethics Committee of the Indian Institute of Technology Ropar (approval number: 01–2016), and all procedures adhered to the Declaration of Helsinki. Sample size was determined based on previous studies on sentence planning [26,27,46,104].

### Stimulus materials and experimental procedure

Participants described 55 line drawings depicting 2-participant (transitive) events, interspersed among 62 line drawings of 1-participant (intransitive) events. S3 Fig shows an example of a 2-participant picture. The order of stimulus presentation was randomized for each participant at runtime. To counterbalance the direction of agent/patient fixations, pictures were presented in 2 lists with vertically mirrored versions and lists contained roughly the same number of pictures with agents on the right or left.

Participants were assigned alternatingly to 1 of 2 groups (25 participants per group) and were instructed to describe the pictures with sentences either in imperfective aspect (as if the event was ongoing, aligned condition) or in perfective aspect (as if the event was completed, nonaligned condition). We chose this between-subject design to avoid priming effects when switching conditions and because we expected planning and encoding processes to be influenced by the repeated presentation of the same picture stimuli [105]. (Participants groups did not differ in working memory capacity and executive function measures, cf. S1 Text, S1 Fig, S2 Table). Participants were instructed to describe the pictures spontaneously, to start speaking as early as possible, and to mention all depicted event participants. The spontaneous elicitation of picture descriptions [25,26] aimed at ensuring that participants planned their sentences in the most natural way possible. While the overall semantic content of responses in such a paradigm is guided by what was depicted, participants’ freedom in how to respond also often leads to responses that do not conform to the target structures. The experiment proceeded in a self-paced manner so that participants initiated the start of the each trial by button press. Participants were given short breaks after approximately every 25 trials. The experiment lasted approximately 35 minutes. Eye tracking data were recorded using a Tobii TX-300 eye tracker (Tobii AB, Stockholm, Sweden) at a sampling rate of 250 Hz. EEG data were recorded with a 129 channel (128 + VREF) HydroCel Geodesic Sensor Net (Electrical Geodesics, Eugene, Oregon, United States of America), at a sampling rate of 500 Hz and amplified by a NET AMPS 400 amplifier.

### Eye tracking analysis

Eye tracking data were processed in R [106]. Fixations were extracted from the eye tracker’s raw samples [107], consecutive fixations to agent and patients in the pictures were subsumed into gazes [108] and, for the analyses, aggregated into 100-ms bins for each trial to reduce temporal autocorrelation [109]. Only transitive sentences in which participants named both agent and patient characters and used the targeted sentence structures were included for the analysis because in intransitives, there is only 1 character to fixate. Trials with response latencies larger than 6 seconds or longer than 2.5 SD from a participant’s mean, as well as trials with first fixations to agent or patient characters later than 500 ms after stimulus onset or with track loss, were excluded. On balance, 1,552 trials were included (57.4% of all trials with transitive picture stimuli). The full exclusion criteria are described in S1 Text.

Based on the literature [25,26,45,46] and visual inspection of the eye tracking graph, we determined the time window of interest to span from 200 to 800 ms after stimulus onset. As expected, very few language-related eye movements were observed before 200 ms [49,110]. Eye fixations to agent characters were analyzed on the single-trial level with logistic mixed-effects growth curve regression [52,111,112]. Based on visual inspection of the number of inflection points of the fixation curves in the analysis time window [52], linear, quadratic, cubic time terms, and their interactions with aligned versus nonaligned conditions were included as predictors. Additional variables and their interactions with the time terms were also included to control for their potential influence on the fixation time course. These were speech onset, naming agreement/codability, noun phrase length, trial number, as well as a number of stimulus properties (full details are given in S1 Text). The models also included random (group-level) effects for participants (intercept and slopes for time terms) and pictures (intercept and slopes for time terms, alignment type, and their interaction). The full model structure is described in S1 Text. Inferences on the statistical significance of predictors are based on likelihood ratio tests. Fixation proportions in Fig 2A were smoothed with simple moving averages over 12 epochs (48 ms).

### Electroencephalography (EEG) analysis

EEG data were preprocessed in EEGLAB (version 14.1.2, [113]), FieldTrip (version 20190107, [114]), and R (version 3.6.0, [106]). Continuous EEG data were re-referenced to the average of the left and right mastoids offline, filtered (0.3 to 40 Hz band-pass filter), and down-sampled to a sampling rate of 250 Hz. Pauses between blocks were removed, and artifactual channels were automatically identified (by deviation of more than 5 SD from the mean of all channels in kurtosis or probability). The data were then decomposed into independent components (excluding rejected channels) and epoched from −1,000 to 1,500 ms relative to stimulus picture onset. The SASICA and ADJUST algorithms [115,116] were used to identify artifactual components (number of rejected independent components: mean = 32.10, SD = 8.00). Previously rejected channels were spherically interpolated after artifactual components were subtracted from the EEG data.

The single-trial data were then transformed into a time–frequency representation (in 0.5-Hz steps) using wavelet decomposition (multi-taper method convolution) with Hanning-tapered time windows using a length of 3 cycles and advancing in 100-ms steps. Power was averaged separately for each frequency across channels into ROIs to reduce spatial autocorrelation. S7 Table shows the grouping of electrodes into ROIs. Power spectra were then transformed into dB relative to median power in a baseline period of −600 to −200 ms before stimulus picture onset in order to mitigate the 1/*f* problem and to make power in all frequencies directly comparable [117]. Finally, power was averaged within frequency bands. Frequency bands were defined individually, based on each participant’s individual peak alpha frequency (IAF) [80]. We extracted IAFs [118] from resting state EEG recordings in which participants sat still with their eyes open and closed for 2 minutes each, recorded once directly before and once directly after the picture description experiment. The theta band was defined as ranging from IAF-6 to IAF-4 (on average 4.12 to 6.12 Hz, *w* = 2.63 to 8.07 Hz). The alpha band was defined as ranging from IAF-4 to IAF+2 (on average 6.12 to 12.12 Hz, *w* = 4.63 to 14.07 Hz). The IAF of 1 participant could not be computed due to excessive artifacts and was imputed with the median IAF of all other participants.

Throughout all analyses, we time-locked to the onset of the picture stimulus because time-locking the neural signals in a way to single out specific steps in the planning of sentences is not feasible. The average timing of these steps is potentially too variable, unlike the average timing of planning steps for the production of single words in isolation, which is well known [119]. This is in contrast to studies on sentence comprehension where the timing of external events (such as the presentation of individual words) can be precisely determined and thus allows the study of evoked potentials.

We included transitive and intransitive sentences in which participants named all picture characters and used the targeted sentence structures. We excluded epochs that were found to be contaminated by heavy artifacts (by visual inspection after independent components correction). To avoid muscle artifacts resulting from movements of the articulators during the 0- to 800-ms analysis time window, only epochs of trials in which participants began speaking later than 1,500 ms after picture onset and that did not contain any “pre-speech noises” (such as smacking lips or saying “uh”) were included. Thus, speech onset occurred at least 700 ms after the end of our analysis time window in all of the included trials. Spoken word encoding requires around 600 ms [119], speakers retrieve depicted characters’ names immediately before uttering them [57] and preparations for speaking affect oscillatory power roughly up to 400 to 500 ms before speech onset [60]. Our analysis time window can therefore be expected to cover primarily relational-structural planning processes of utterances in which participants started speaking at 1,500 ms or later. On balance, 3,447 trials were included in the EEG analysis (58.9% of all trials).

The time course of power changes in the EEG theta and alpha frequency bands was analyzed with linear mixed-effects regression model trees as implemented in the R packages glmertree [53] and lme4 [112]. Regression model trees recursively partitioned the data into subsets to find the best-fitting model in each cell of these partitions, based on which subgroups showed statistically similar effects of ROI or sentence type (Figs 1 and 3A) or their interaction. The fixed effects structure was a fourth-order growth curve model on the time course of EEG power in a time window from 0 to 800 ms, analogously to the time window in the eye tracking analysis. The EEG time window started at 0 ms because electrophysiological responses to stimulus onset can occur almost immediately (unlike eye fixations). The choice of the order of polynomials was based on visual inspection of the shapes of the power curves. Subgroups in the tree were identified by their behavior with respect to the estimates for the regressors (intercept and polynomial time terms). On each iteration, the regression tree algorithm searched for differences in the robustness of model estimates (polynomial time courses, across ROIs and sentence types, conditioned by random effects; technically known as a parameter instabilities [53,120]). The data were then partitioned along the variable for which a split led to the greatest increase in fit on that iteration [120], i.e., for which the largest significant differences between subgroups were identified.

Random effects (intercepts and slopes for time terms by participant and by picture) and fixed effects of additional control variables were fitted globally. Analogously to the eye tracking model, the model included as fixed effects control variables of speech onset, naming agreement/codability, noun phrase length, trial number, as well as a number of stimulus properties (see S1 Text). Inferences on the statistical significance of splits were assessed through parameter instability tests during the tree fitting process [53,120].

Time courses of power changes in Figs 2B and 3B are smoothed with local polynomial regression (loess) with a span of 0.4. To visualize the extent of the observed differences in event-related power changes between aligned and nonaligned sentences for the theta band and between sentences with marked and unmarked noun phrases for the alpha band, we also plotted topographic maps with *t*-values (Figs 2D and 3D) across all scalp electrodes. For this plot, mean power during −600 ms to −200 ms relative to picture onset served as baseline.

## Supporting information

S1 Fig Individual differences measures for the 2 experimental groups, instructed to describe pictures in imperfective aspect (transitive sentences are aligned with intransitive sentences) vs. perfective aspect (transitive sentences are not aligned with intransitive sentences). (A) Mean partial-credit load scores for automated complex span tasks. (B) Mean congruency effect for Flanker task. (Underlying data and scripts are available from https://osf.io/uhtcn/ and in the Supporting information file S1 Data.)
(TIFF)

S2 Fig Distribution of speech onset latencies for intransitive (1 participant) and transitive (2 participants) sentences in the aligned (ongoing events in imperfective aspect) and nonaligned (completed events in perfective aspect) conditions. Speech onset latencies did not significantly differ between conditions (all *p*s > 0.13, see S3 Table). (Underlying data and scripts are available from https://osf.io/uhtcn/ and in the Supporting information file S1 Data.)
(TIFF)

S3 Fig Example 2-participant (transitive) stimulus picture. (A) Line drawing showing an agent (“gardener”) performing an action (“planting”) on a patient (“tree”); however, picture versions with gray backgrounds were presented during the experiment. (B) Scrambled gray background version of the stimulus picture with all pixels randomly redistributed over the screen display and black fixation square. (C) Gray background version of example stimulus.
(TIFF)

S4 Fig Model tree for power changes (in dB) in individually defined theta frequency bands between 0 and 800 ms, showing the subsidiary splits. Scalp maps show electrode positions of ROIs included in the respective grouping (see S5 Table for details). All splits were statistically significant with *p*<0.003. Tree tips show model fits for the respective grouping; ribbons represent 95% CIs. (Underlying data and scripts are available from https://osf.io/uhtcn/ and in the Supporting information file S1 Data.) ROI, region of interest.
(TIFF)

S5 Fig Model tree for power changes (in dB) in individually defined alpha frequency bands between 0 and 800 ms, showing the subsidiary split between intransitive and transitive sentences with nominative marking in posterior ROIs. Scalp maps show electrode positions of ROIs included in the respective grouping (see S5 Table for details). All splits were statistically significant with *p*<.02. Tree tips show model fits for the respective grouping; ribbons represent 95% CIs. (Underlying data and scripts are available from https://osf.io/uhtcn/ and in the Supporting information file S1 Data.) ROI, region of interest.
(TIFF)

S1 Table Example sentences from Hindi illustrating the split-ergative case marking system. More literal translations are “The gardener is crouching/had crouched” and “The gardener is planting a tree/had planted a tree.” Sentence types were chosen so that the overall syntactic structure matched as much as possible across conditions and all sentences consisted of 1 or 2 noun phrases, a verb, and an auxiliary. AUX, auxiliary; ERG, ergative case; IPFV, imperfective aspect; NOM, nominative case; PFV, perfective aspect; ∅, null (phonologically empty) morpheme.
(PDF)

S2 Table Mean partial-credit load scores for automated complex span tasks and mean congruency effect for Flanker task (standard deviations in parentheses). (Underlying data and scripts used to generate graphs are available from https://osf.io/uhtcn/.)
(PDF)

S3 Table Generalized gamma linear mixed-effects regression results modeling speech onset latencies (inverse link function). Statistical significance of predictors was assessed using likelihood ratio tests; significance was only assessed for the predictors of interest (sentence transitivity and alignment condition). (Underlying data, scripts, and models are available from https://osf.io/uhtcn/).
(PDF)

S4 Table Generalized (binomial) linear mixed-effects regression results modeling fixations on agent characters (successes) vs. fixations elsewhere in the pictures (failures) between 200 and 800 ms relative to picture stimulus onset. Statistical significance of predictors was assessed using likelihood ratio tests; significance was only assessed for predictors of interest. (Underlying data, scripts, and models are available from https://osf.io/uhtcn/.)
(PDF)

S5 Table Linear mixed-effects regression tree results modeling EEG power dynamics (in dB) in individually defined theta frequency bands. Splits and predicted power time courses are shown in S4 Fig. (Underlying data, scripts, and models are available from https://osf.io/uhtcn/). EEG, electroencephalography.
(PDF)

S6 Table Linear mixed-effects regression tree results modeling EEG power dynamics (in dB) in individually defined alpha frequency bands. Splits and predicted power time courses are shown in S5 Fig. (Underlying data, scripts, and models are available from https://osf.io/uhtcn/). EEG, electroencephalography.
(PDF)

S7 Table Grouping of electrodes of the HydroCel Geodesic Sensor Net into ROIs used for analysis of EEG event-related synchronization/de-synchronization, based on locations represented in FieldTrip; cf. the channel map provided by the manufacturer for another representation of channel locations (https://drive.google.com/file/d/0B388xdH0Vxl2T3U4aS1oWDdPdTg/view). EEG, electroencephalography; ROI, region of interest.
(PDF)

S1 Data Data underlying the plots in Figs 2 and 3 and S1–S5 Figs. (XLSX)

S1 Text Supporting methods. (PDF)

## Abbreviations

EEG: electroencephalography
ERD: event-related desynchronization
ERS: event-related synchronization
IAF: individual alpha frequency
ROI: region of interest
