# Supplementary material for: Neural signatures of syntactic variation in speech planning
Source: PLoS Biol. 2021 Jan 26;19(1):e3001038. doi: 10.1371/journal.pbio.3001038 (PMC7837500; doi:10.1371/journal.pbio.3001038)
Supplement: S1 Table — More literal translations are “The gardener is crouching/had crouched” and “The gardener is planting a tree/had planted a tree.” Sentence types were chosen so that the overall syntactic structure matched as much as possible across conditions and all sentences consisted of 1 or 2 noun phrases, a verb, and an auxiliary. AUX, auxiliary; ERG, ergative case; IPFV, imperfective aspect; NOM, nominative case; PFV, perfective aspect; ∅, null (phonologically empty) morpheme. (PDF) [file pbio.3001038.s006.pdf]

	Agent (Subject)	Patient (Object)	Verb		Apect
intransitive, one-participant events	*mālī* ∅		*jhuktā*	*hai*	
	gardener NOM		crouch:IPFV	AUX	imperfective
	“The gardener crouches.”				
	mālī ∅		*jhukā*	*thā*	
	gardener NOM		crouch:PFV	AUX	perfective
	“The gardener crouched.”				
transitive, two-participant events	*mālī* ∅	*paudhe* ∅	*lagātā*	*hai*	
	gardener NOM	trees NOM	plant:IPFV	AUX	imperfective
	“The gardener plants trees.”				
	mālī ***ne***	*paudhe* ∅	*lagāye*	*the*	
	gardener **ERG**	trees NOM	plant:PFV	AUX	perfective
	“The gardener planted trees.”				

Table S1: Example sentences from Hindi illustrating the split-ergative case marking system. More literal translations are “The gardener is crouching / had crouched” and “The gardener is planting a tree / had planted a tree”. Sentence types were chosen so that the overall syntactic structure matched as much as possible across conditions and all sentences consisted of one or two noun phrases, a verb and an auxiliary. Abbreviations: AUX = auxiliary, ERG = ergative case, IPFV = imperfective aspect, NOM = nominative case, PFV = perfective aspect, ∅ = null (phonologically empty) morpheme.
