# Supplementary material for: Neural signatures of syntactic variation in speech planning
Source: PLoS Biol. 2021 Jan 26;19(1):e3001038. doi: 10.1371/journal.pbio.3001038 (PMC7837500; doi:10.1371/journal.pbio.3001038)
Supplement: S2 Table — (Underlying data and scripts used to generate graphs are available from https://osf.io/uhtcn/.) (PDF) [file pbio.3001038.s007.pdf]

Experimental group	Operation span	Symmetry span	Flanker congruency effect
Aligned condition	45.84 (4.20)	21.60 (4.07)	78.69 (71.48)
Non-aligned condition	44.08 (5.13)	21.76 (4.52)	64.97 (34.75)

Table S2: Mean partial-credit load scores for automated complex span tasks and mean congruency effect for Flanker task (standard deviations in parentheses). (Underlying data and scripts used to generate graphs are available from `https://osf.io/uhtcn/`.)
