# Supplementary material for: Neural signatures of syntactic variation in speech planning
Source: PLoS Biol. 2021 Jan 26;19(1):e3001038. doi: 10.1371/journal.pbio.3001038 (PMC7837500; doi:10.1371/journal.pbio.3001038)
Supplement: S3 Table — Statistical significance of predictors was assessed using likelihood ratio tests; significance was only assessed for the predictors of interest (sentence transitivity and alignment condition). (Underlying data, scripts, and models are available from https://osf.io/uhtcn/). (PDF) [file pbio.3001038.s008.pdf]

Speech onset latencies				
	dependent variable: speech onset latency (in ms); random intercept and random slope for sentence transitivity by participant and random intercept and random slope for aspect by stimulus picture			
	Estimate	$\|t\|$	χ^2	p
Intercept	0.474	21.703		
Alignment Condition (= non-aligned)	−0.039	1.517	2.190	0.139
Sentence Transitivity (= transitive)	−0.031	1.235	1.007	0.316
Alignment Condition × Sentence Transitivity	0.016	0.946	0.885	0.347
Agent NP Length (standardized)	> -0.001	0.066		
Trial Number (standardized)	−0.006	3.772		
Verb Codability (H standardized)	−0.017	1.601		
Visual Picture Complexity (standardized)	−0.002	0.260		

Table S3: Generalized Gamma linear mixed effects regression results modeling speech onset latencies (inverse link function). Statistical significance of predictors was assessed using likelihood ratio tests; significance was only assessed for the predictors of interest (sentence transitivity and alignment condition). (Underlying data, scripts and models are available from `https://osf.io/uhtcn/`.)
