# Supplementary material for: Neural signatures of syntactic variation in speech planning
Source: PLoS Biol. 2021 Jan 26;19(1):e3001038. doi: 10.1371/journal.pbio.3001038 (PMC7837500; doi:10.1371/journal.pbio.3001038)
Supplement: S4 Table — Statistical significance of predictors was assessed using likelihood ratio tests; significance was only assessed for predictors of interest. (Underlying data, scripts, and models are available from https://osf.io/uhtcn/.) (PDF) [file pbio.3001038.s009.pdf]

Agent fixations, 200–800 ms relative to picture onset				
	dependent variable: log odds of fixating on agent character (hits vs. misses)			
	Estimate	$\|z\|$	χ^2	p
Intercept	0.693	3.362		
Linear Time (Time^1)	−1.387	3.854		
Quadratic Time (Time^2)	0.455	1.731		
Cubic Time (Time^3)	−0.489	2.272		
Alignment (= non-aligned)	0.041	0.397	0.191	0.662
Alignment × Time^1	−0.042	0.158	0.016	0.899
Alignment × Time^2	−0.438	1.841	3.109	0.078
Alignment × Time^3	1.133	4.442	16.817	< 0.001
Speech Onset (standardized)	−0.005	0.532		
Speech Onset × Time^1	0.015	0.612		
Speech Onset × Time^2	−0.009	0.382		
Speech Onset × Time^3	0.140	5.572		
Agent NP Length (standardized)	−0.053	4.875		
Agent NP Length × Time^1	0.065	2.678		
Agent NP Length × Time^2	0.069	2.748		
Agent NP Length × Time^3	−0.091	3.494		
Trial Number (standardized)	−0.078	10.899		
Trial Number × Time^1	−0.064	3.932		
Trial Number × Time^2	0.004	0.222		
Trial Number × Time^3	−0.006	0.335		
Size Agent AOI (standardized)	0.288	2.592		
Size Patient AOI (standardized)	−0.443	3.956		
Agent Humanness (= non-human)	−0.498	2.129		
Agent Codability (H standardized)	−0.021	0.194		
Verb Codability (H standardized)	0.191	1.446		
Visual Picture Complexity (standardized)	0.038	0.321		
Autocorrelation at Lag = 1 (agent successes, centered)	0.191	194.607		

Table S4: Generalized (binomial) linear mixed effects regression results modeling fixations on agent characters (successes) vs. fixations elsewhere in the pictures (failures) between 200 and 800 ms relative to picture stimulus onset. Statistical significance of predictors was assessed using likelihood ratio tests; significance was only assessed for predictors of interest. (Underlying data, scripts and models are available from `https://osf.io/uhtcn/`.)
