# Supplementary material for: Neural signatures of syntactic variation in speech planning
Source: PLoS Biol. 2021 Jan 26;19(1):e3001038. doi: 10.1371/journal.pbio.3001038 (PMC7837500; doi:10.1371/journal.pbio.3001038)
Supplement: S5 Table — Splits and predicted power time courses are shown in S4 Fig. (Underlying data, scripts, and models are available from https://osf.io/uhtcn/). EEG, electroencephalography. (PDF) [file pbio.3001038.s010.pdf]

Table S5: Linear mixed effects regression tree results modeling EEG power dynamics (in dB) in individually defined theta frequency bands. Splits and predicted power time courses are shown in Fig S4. Underlying data, scripts and models are available from `https://osf.io/uhtcn/`.

Theta-band power dynamics, 0–800 ms relative to picture onset			
		dependent variable: dB	
		Estimate	Standard error
Global			
	Intercept	0.711	0.049
	Speech Onset (standardized)	−0.010	0.007
	Speech Onset × $Time^1$	0.010	0.020
	Speech Onset × $Time^2$	−0.017	0.020
	Speech Onset × $Time^3$	−0.056	0.020
	Speech Onset × $Time^4$	0.035	0.020
	Agent NP Length (standardized)	−0.009	0.007
	Agent NP Length × $Time^1$	0.013	0.020
	Agent NP Length × $Time^2$	−0.011	0.020
	Agent NP Length × $Time^3$	0.099	0.020
	Agent NP Length × $Time^4$	0.033	0.020
	Trial Number (standardized)	0.025	0.005
	Trial Number × $Time^1$	−0.010	0.016
	Trial Number × $Time^2$	−0.033	0.016
	Trial Number × $Time^3$	0.029	0.016
	Trial Number × $Time^4$	0.049	0.016
	Number of Fixations (standardized)	−0.012	0.006
	Number of Fixations × $Time^1$	0.037	0.018
	Number of Fixations × $Time^2$	−0.005	0.018
	Number of Fixations × $Time^3$	−0.017	0.018
	Number of Fixations × $Time^4$	0.083	0.018
	Agent Codability (H standardized)	< 0.001	0.019
	Verb Codability (H standardized)	0.009	0.019
	Agent Humanness (= non-human)	−0.025	0.025
	dB at Lag = 1 (centered)	0.771	0.001
Tree-tip specific			
Frontal & left-mid central, aligned	$Time^1$	−0.572	0.133
	$Time^2$	0.109	0.105
	$Time^3$	0.448	0.117
	$Time^4$	−0.368	0.123

Table S5 continued from previous page

Frontal & left-mid central, non-aligned, intransitive	Intercept	−0.075	0.056
	$Time^1$	−0.432	0.136
	$Time^2$	0.074	0.109
	$Time^3$	0.285	0.121
	$Time^4$	−0.069	0.127
Frontal & left-mid central, non-aligned, transitive	Intercept	−0.160	0.057
	$Time^1$	−0.434	0.139
	$Time^2$	0.111	0.112
	$Time^3$	0.366	0.124
	$Time^4$	−0.214	0.129
Posterior & right central, aligned, intransitive	Intercept	0.079	0.018
	$Time^1$	−0.751	0.137
	$Time^2$	0.204	0.110
	$Time^3$	0.772	0.122
	$Time^4$	−0.663	0.128
Posterior & right central, aligned, transitive	Intercept	0.084	0.019
	$Time^1$	−0.930	0.139
	$Time^2$	0.132	0.112
	$Time^3$	0.601	0.124
	$Time^4$	−0.581	0.130
Right central, non-aligned	Intercept	−0.072	0.059
	$Time^1$	−0.679	0.146
	$Time^2$	0.131	0.121
	$Time^3$	0.496	0.131
	$Time^4$	−0.374	0.137
Posterior, non-aligned	Intercept	0.056	0.056
	$Time^1$	−0.831	0.135
	$Time^2$	0.321	0.108
	$Time^3$	0.507	0.120
	$Time^4$	−0.473	0.125
