# Supplementary material for: Neural signatures of syntactic variation in speech planning
Source: PLoS Biol. 2021 Jan 26;19(1):e3001038. doi: 10.1371/journal.pbio.3001038 (PMC7837500; doi:10.1371/journal.pbio.3001038)
Supplement: S6 Table — Splits and predicted power time courses are shown in S5 Fig. (Underlying data, scripts, and models are available from https://osf.io/uhtcn/). EEG, electroencephalography. (PDF) [file pbio.3001038.s011.pdf]

Table S6: Linear mixed effects regression tree results modeling EEG power dynamics (in dB) in individually defined alpha frequency bands. Splits and predicted power time courses are shown in Fig S5. (Underlying data, scripts and models are available from `https://osf.io/uhtcn/`)

Alpha-band power dynamics, 0–800 ms relative to picture onset		dependent variable: dB	
		Estimate	Standard error
Global			
	Intercept	−0.236	0.076
	Speech Onset (standardized)	0.009	0.008
	Speech Onset × $Time^1$	0.022	0.020
	Speech Onset × $Time^2$	−0.028	0.021
	Speech Onset × $Time^3$	−0.009	0.021
	Speech Onset × $Time^4$	0.024	0.022
	Agent NP Length (standardized)	−0.014	0.008
	Agent NP Length × $Time^1$	0.000	0.019
	Agent NP Length × $Time^2$	−0.047	0.022
	Agent NP Length × $Time^3$	0.082	0.022
	Agent NP Length × $Time^4$	0.014	0.022
	Trial Number (standardized)	0.017	0.006
	Trial Number × $Time^1$	−0.012	0.017
	Trial Number × $Time^2$	−0.021	0.017
	Trial Number × $Time^3$	−0.020	0.017
	Trial Number × $Time^4$	0.005	0.017
	Number of Fixations (standardized)	0.013	0.007
	Number of Fixations × $Time^1$	−0.032	0.019
	Number of Fixations × $Time^2$	0.032	0.019
	Number of Fixations × $Time^3$	−0.036	0.019
	Number of Fixations × $Time^4$	−0.060	0.019
	Agent Codability (H standardized)	0.008	0.023
	Verb Codability (H standardized)	−0.003	0.023
	Agent Humanness (= non-human)	> -0.001	0.030
	dB at Lag = 1 (centered)	0.595	0.002
Tree-tip specific			
Frontal, unmarked	$Time^1$	−0.423	0.103
	$Time^2$	0.211	0.087
	$Time^3$	0.356	0.089
	$Time^4$	−0.635	0.111

Table S6 continued from previous page

Frontal,	Intercept	0.029	0.030
marked	$Time^1$	−0.288	0.119
	$Time^2$	0.107	0.108
	$Time^3$	0.179	0.110
	$Time^4$	−0.635	0.111
Central,	Intercept	−0.060	0.015
unmarked	$Time^1$	−0.687	0.103
	$Time^2$	0.225	0.087
	$Time^3$	0.423	0.089
	$Time^4$	−0.383	0.090
Posterior,	Intercept	−0.014	0.024
unmarked,	$Time^1$	−0.795	0.115
transitive	$Time^2$	0.229	0.102
	$Time^3$	0.645	0.104
	$Time^4$	−0.599	0.105
Posterior,	Intercept	−0.081	0.017
unmarked,	$Time^1$	−0.932	0.105
intransitive	$Time^2$	0.134	0.091
	$Time^3$	0.639	0.093
	$Time^4$	−0.454	0.094
Posterior,	Intercept	0.024	0.026
marked	$Time^1$	−0.641	0.110
	$Time^2$	0.200	0.099
	$Time^3$	0.324	0.101
	$Time^4$	−0.570	0.102
