# Supplementary material for: Neural signatures of syntactic variation in speech planning
Source: PLoS Biol. 2021 Jan 26;19(1):e3001038. doi: 10.1371/journal.pbio.3001038 (PMC7837500; doi:10.1371/journal.pbio.3001038)
Supplement: S7 Table — EEG, electroencephalography; ROI, region of interest. (PDF) [file pbio.3001038.s012.pdf]

Region of interest (ROI)	Electrodes	Electrode positions
left frontal	E20 E23 E24 E27 E28 E29 E34 E35	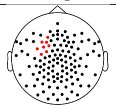
mid frontal	E4 E5 E10 E11 E12 E16 E18 E19	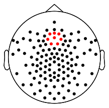
right frontal	E3 E110 E111 E116 E117 E118 E123 E124	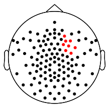
left central	E30 E36 E37 E40 E41 E42 E46 E47	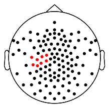
mid central	E6 E7 E13 E31 E54 E55 E79 E80 E106 E112 Cz	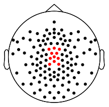
right central	E87 E93 E98 E102 E103 E104 E105 E109	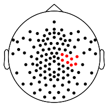
left posterior	E51 E52 E53 E58 E59 E60 E65 E66	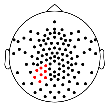
mid posterior	E61 E62 E67 E72 E71 E77 E76 E78	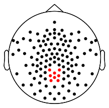
right posterior	E84 E85 E86 E90 E91 E92 E96 E97	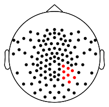

Table S7: Grouping of electrodes of the HydroCel Geodesic Sensor Net into regions of interest used for analysis of EEG event-related spectral perturbations, based on locations represented in FieldTrip; cf. the channel map provided by the manufacturer for another representation of channel locations (`https://drive.google.com/file/d/0B388xdH0Vxl2T3U4aS1oWDdPdTg/view`).
