# Supplementary material for: Neural signatures of syntactic variation in speech planning
Source: PLoS Biol. 2021 Jan 26;19(1):e3001038. doi: 10.1371/journal.pbio.3001038 (PMC7837500; doi:10.1371/journal.pbio.3001038)
Supplement: S1 Text — (PDF) [file pbio.3001038.s014.pdf]

Supporting methods

Working memory capacity and inhibitory control measures

To ensure that there were no differences in executive functioning [8, 33] between the experimental groups, we measured working memory capacity and inhibitory control during a second experimental session of approximately 40 minutes for which participants returned to the laboratory a few days after the sentence production experiment. Participants completed automated, shortened versions of the operation span and symmetry span tasks [12, 27] and a Flanker task [10]. The order of tasks was balanced across participants. We operationalized participants' working memory capacity as the partial-credit load scores [6], i.e., the total number of correctly recalled elements across all items. Inhibitory control was operationalized as the congruency effect [20] for each participant, i.e., the difference between the mean reaction time on incongruent trials and the mean reaction time on congruent trials. During the Flanker task, fifty congruent, incongruent, and neutral trials were administered, respectively. The results of the executive function measures are presented in Table S2 and Fig S1. We failed to reject the null hypothesis of no difference in both the working memory capacity measures (mean partial-credit load scores: operation span, $t(46.197) = 1.327$, $p = 0.19$; symmetry span, $t(47.485) = -0.131$, $p = 0.90$), and in inhibitory control (mean congruency effect, $t(34.747) = 0.863$, $p = 0.39$).

Reaction time analyses

Experimental participants might have needed more time to perform an additional cognitive operation when describing completed events (perfective aspect) regardless of the difference of interest (non-aligned sentences). The picture stimuli mostly showed events in the middle of the action. This might have required that speakers first had to transform their mental representation from an ongoing event to a completed event. By contrast, when planning sentences in imper-

fective aspect (describing ongoing events) no such additional transformation is needed.

In order to evaluate this possibility, we compared speech onset latencies between sentences produced in perfective and imperfective aspect to test whether there are behavioral differences in when speakers started articulating their utterances, depending on whether the event is described as already being completed or as ongoing.

In the analysis we included all and only trials in which participants produced intransitive or transitive sentences in imperfective or perfective aspect and overtly named all event participants conforming to the instructions. We excluded trials with speech onset latencies later than 6000 ms or longer than 2.5 SD away from each participant's mean speech onset latency. On balance, 4066 trials (68.1%) were included in the analysis.

Speech onset latencies were modelled with Gamma regression [18] with sentence transitivity and alignment condition (aligned/imperfective aspect vs. non-aligned/perfective aspect) as critical predictors and length of the subject NP (in syllables), trial number, verb codability and visual picture complexity (number of black pixels) as control variables (cf. section on statistical analyses below). A random intercept by participant with a random slope for sentence transitivity and a random intercept by stimulus picture with a random slope for alignment condition were included; this constitutes the maximal random effects structure in the study's between-participants design [3]. We failed to reject the null hypothesis of no differences between intransitive and transitive sentences or between non-aligned/perfective aspect and aligned/imperfective aspect sentences (all $p > 0.13$, Table S3, Fig S2). We conclude that any differences in speakers' task demands in the two alignment conditions are unlikely to have lead to the effects we observed.

Details on experimental procedure

Experimental sessions started with participants giving informed consent and reading the instructions (in Hindi). The experimenter then answered any questions about the procedure and mounted the EEG cap and calibrated the eye tracker. Participants first read a summary of the instructions on the screen again before starting a practice block in which they saw example pictures accompanied by prerecorded example descriptions that demonstrated how intran-

sitive and transitive pictures could be described in imperfective or perfective aspect. Next, the same pictures were presented again in a different order and participants were asked to describe them spontaneously themselves.

Experimental trials started with the presentation of a scrambled version of the trial's stimulus picture with a superimposed fixation square. This square was positioned randomly in one out of five positions on the top of the screen (left, left-middle, middle, right-middle, right) for a jittered interval between 1750 and 2250 ms (serving as baseline period). The fixation square was positioned at the top of the screen to avoid that participants' gaze already fell on one of the event participants when the stimulus picture appeared [13, 21, 22, 25]. Scrambled versions of the stimulus pictures were used during presentation of the fixation square to make the luminosity of this display similar to the display of the stimulus picture without providing an actual preview of the stimulus [17]. To produce the scrambled pictures, pixels were randomly redistributed over the screen (cf. Fig S3). Following the fixation square display, the stimulus picture appeared and participants described it, ending the trial with a button press on a response pad after they finished speaking.

E-Prime 2.0 (Psychology Software Tools, Sharpsburg) was used to present stimuli and control the data recordings. Stimulus pictures were presented at a distance of approximately 60 cm on the screen of a Tobii TX-300 eye tracker (Tobii AB, Stockholm; refresh rate = 60 Hz) with a resolution of 1920 × 1080 pixels. The pictures exhibited a grey background (hex triplet: B9B9B9) to reduce the overall contrast between the drawing's black lines and the background.

The impedance of the EEG was kept below 50 kΩ, following the manufacturer's recommendation. Vocal responses were recorded using a microphone positioned next to the screen and directed at the participant.

Details on data preprocessing

Vocal responses were transcribed and annotated with additional information. This information included: (a) the order of words, (b) the words speakers chose to describe characters and actions, (c) case marking on nouns, (d) the aspect of the verb, and (e) whether the speakers corrected themselves or started over.

Eye tracking data were processed in R [26]. Fixations were detected in the raw samples from the eye tracker [9, 32] and subsumed in gazes containing all consecutive fixations on one area of interest [15], making the fixation data

a measure of visual attention by interpolating saccades between fixation locations on the same object on the screen. For each sample in each trial, we then calculated whether visual attention fell on one of the defined areas of interest (agent and patient). Fixation samples were aggregated into 100 ms bins for each trial to reduce the temporal auto-correlation between consecutive fixation samples as eye movements are much slower than the eye tracker's sampling rate [1, 5, 16]. We included only transitive sentences in response to the presentation of two-participant pictures in the eye tracking analysis because in intransitive, one-participant pictures only one character is available to be fixated. This restricts the possibilities for fixations that are not directed towards the subject character.

Electrophysiological data were processed in MATLAB (The Mathworks, Natick MA) with the EEGLAB [7], ERPLAB [19] and FieldTrip [23] toolboxes and in R. Before transformation into dB relative to the baseline period, outlier power values were identified for each participant across all regions of interest (lowest and highest 2.5% of values, respectively) and linearly interpolated.

In the eye tracking analysis, only trials in which participants described two-participant stimulus pictures with grammatical, transitive sentences with overtly mentioned agents, patients and verbs with agent-patient-verb ("SOV") word order in either perfective or imperfective aspect were included. We excluded sentences in other aspects (e.g., continuous) from the analysis. Perfective transitive sentences in which the agent NP did not carry ergative case marking (postposition *ne*) or imperfective sentences with accusative-marked subjects were excluded, as were responses with speech onsets after presentation of the stimulus picture that were longer than 6000 ms or more than 2.5 SD longer than a participant's mean speech onset latency. Trials were also excluded if participants corrected themselves or started over with their description. Pauses or disfluencies, however, were tolerated because they are a natural feature of language use. For the eye tracking analysis, we additionally excluded trials in which the participants did not look at the fixation dot at stimulus onset. We did this to preclude that planning processes were influenced simply by whichever character happened to be fixated at that time [13, 21]. Trials in which the first fixation on the agent or the patient occurred later than 500 ms after stimulus picture onset and trials with track loss (defined as a gap of more than 600 ms between two consecutive fixations) were excluded, as were trials in which participant never fixated on either the agent or the patient character.

In the EEG analysis, we also included intransitive sentences with overtly

mentioned argument and verbs with SV word order in either perfective or imperfective aspect. The structural exclusion criteria were the same as for the eye tracking analysis. We additionally excluded epochs that were found (upon visual inspection) to be contaminated by heavy artifacts. To avoid muscle artifacts resulting from movements of the articulators during the 0–800 ms analysis time window, only epochs of trials in which participants began speaking later than 1500 ms after picture onset and that did not contain any "pre-speech noises" (such as smacking lips or saying "uh") were included. One participant was replaced because no audio was recorded from the participant's vocal responses due to malfunctioning recording equipment.

Details on statistical analyses

In the generalized mixed effects regression for the eye tracking data, a number of control predictors were included to accommodate their potential influence [28] on fixations to the agents in the pictures:

- *Speech onset latency* to capture effects on the fixation curves that are due to faster or slower planning. Speech onset latency also served as a general predictor of planning effort (main effect and interactions with time terms).
- *The codability of agents/subjects and verbs*, reflecting how much participants agreed on how to spontaneously name the characters and the depicted actions to indicate the ease of naming [14, 31] (main effect). Codability was measured by calculating the Shannon entropy H [30] of naming choices for agent and verb in the trials included in the analyses, where a lower value of H means that speakers agreed to a higher degree on which names and words to use than a higher value of H.
- *Total length in syllables of the agent noun phrase*, including any modifiers (e.g., adjectives) that participants might have produced to account for the fact that non-aligned agent phrases that include the ergative case marker *ne* are inherently longer than aligned (nominative) agents (main effect and interactions with time terms). It is important to note, however, that agent phrase lengths also differed within aligned and within non-aligned sentences because participants labeled the characters spontaneously with names consisting of one or more syllables.

- *Trial number* to capture possible syntactic priming effects [24], as well as fatigue and training effects over the course of the experiment (main effect and interactions with time terms).
- *Properties of the stimulus pictures* that might have influenced eye movements were captured by including as predictors the visual complexity of the picture (measured as the number of black pixels, assuming that more complex pictures contain more lines and shapes), the size of the agent/subject and patient areas of interest (in percent coverage of the screen) and the humanness of the agent characters (main effects).

The number of fixation hits on the agent in each respective previous time bin was included as a predictor to control for temporal auto-correlation in the eye movement signal [1, 5, 29]. Categorical predictors were deviation coded (-0.5, 0.5). Continuous predictors were z-transformed. We used the maximal random effects structure justified by design [2, 3]. No random effects for control predictors were included [3]. Models were fit with the *lme4* package [4] in R [26]. The full model output is shown in Table S4.

The tree-based algorithm used for modelling power changes [11] also allows the inclusion of control variables (like in the eye tracking analysis). These were:

- *Speech onset latency* (main effect and interactions with time terms)
- *Agent/subject and verb codability* (main effect)
- *Length of the agent phrase* in syllables (main effect and interactions with time terms)
- *Trial number* (main effect and interactions with time terms)
- *Agent humanness* (main effect)
- *Number of saccades* that were initiated during the analysis time window to account for effects of potential remnants of eye movement artifacts in the data (main effect and interactions with time terms).

Temporal auto-correlation was taken into account by including the power value at the previous time step as a predictor. Crossed random effects for participants and stimuli were included with random intercepts and slopes for the polynomial time terms. The random slope for linear time by stimuli was dropped for the alpha band model to allow model convergence. Control variables and

random effects were estimated globally. Categorical predictors were treatment coded (0, 1). Continuous predictors were z-transformed.

References

[1] D. J. Barr. Analyzing 'visual world' eyetracking data using multilevel logistic regression. *Journal of Memory and Language*, 59(4):457–474, 2008.

[2] D. J. Barr. Random effects structure for testing interactions in linear mixed-effects models. *Frontiers in Psychology*, 4(328), 2013.

[3] D. J. Barr, R. Levy, C. Scheepers, and H. J. Tily. Random effects structure for confirmatory hypothesis testing: Keep it maximal. *Journal of Memory and Language*, 68(3):255–278, 2013.

[4] D. Bates, M. Mächler, B. Bolker, and S. Walker. Fitting linear mixed-effects models using lme4. *Journal of Statistical Software*, 67(1):1–48, 2015.

[5] S.-J. Cho, S. Brown-Schmidt, and W.-y. Lee. Autoregressive generalized linear mixed effect models with crossed random effects: An application to intensive binary time series eye-tracking data. *Psychometrika*, 83(3):751–771, 2018.

[6] A. R. A. Conway, M. J. Kane, M. F. Bunting, D. Z. Hambrick, O. Wilhelm, and R. W. Engle. Working memory span tasks: A methodological review and user's guide. *Psychonomic Bulletin & Review*, 12(5):769–786, 2005.

[7] A. Delorme and S. Makeig. EEGLAB: an open source toolbox for analysis of single-trial EEG dynamics including independent component analysis. *Journal of Neuroscience Methods*, 134(1):9–21, 2004.

[8] A. Diamond. Executive functions. *Annual Review of Psychology*, 64:135–168, 2013.

[9] R. Engbert and R. Kliegl. Microsaccades uncover the orientation of covert attention. *Vision Research*, 43(9):1035–1045, 2003.

[10] B. A. Eriksen and C. W. Eriksen. Effects of noise letters upon the identification of a target letter in a nonsearch task. *Perception & Psychophysics*, 16(1):143–149, 1974.

[11] M. Fokkema, N. Smits, A. Zeileis, T. Hothorn, and H. Kelderman. Detecting treatment-subgroup interactions in clustered data with generalized linear mixed-effects model trees. *Behavior Research Methods*, 50(5):2016–2034, 2018.

[12] J. L. Foster, Z. Shipstead, T. L. Harrison, K. L. Hicks, T. S. Redick, and

R. W. Engle. Shortened complex span tasks can reliably measure working memory capacity. *Memory & Cognition*, 43(2):226–236, 2015.

[13] L. R. Gleitman, D. January, R. Nappa, and J. C. Trueswell. On the *give* and *take* between event apprehension and utterance formulation. *Journal of Memory and Language*, 57(4):544–596, 2007.

[14] Z. M. Griffin. Gaze durations during speech reflect word selection and phonological encoding. *Cognition*, 82(1):B1–B14, 2001.

[15] Z. M. Griffin and J. C. Davison. A technical introduction to using speakers' eye movements to study language. *The Mental Lexicon*, 6(1):53–82, 2011.

[16] K. Holmqvist, M. Nyström, R. Andersson, R. Dewhurst, H. Jarodzka, and J. van de Weijer. *Eye tracking: A comprehensive guide to methods and measures*. Oxford University Press, Oxford, 2011.

[17] F. Huettig, J. Rommers, and A. S. Meyer. Using the visual world paradigm to study language processing: A review and critical evaluation. *Acta Psychologica*, 137(2):151–171, 2011.

[18] S. Lo and S. Andrews. To transform or not to transform: using generalized linear mixed models to analyse reaction time data. *Frontiers in Psychology*, 6(1171), 2015.

[19] J. Lopez-Calderon and S. J. Luck. ERPLAB: an open-source toolbox for the analysis of event-related potentials. *Frontiers in Human Neuroscience*, 8(213), 2014.

[20] J. C. Mullane, P. V. Corkum, R. M. Klein, and E. McLaughlin. Interference control in children with and without ADHD: A systematic review of flanker and simon task performance. *Child Neuropsychology*, 15(4):321–342, 2009.

[21] A. Myachykov, S. Garrod, and C. Scheepers. Determinants of structural choice in visually situated sentence production. *Acta Psychologica*, 141(3):304–315, 2012.

[22] E. Norcliffe and A. E. Konopka. Vision and language in cross-linguistic research on sentence production. In R. K. Mishra, N. Srinivasan, and F. Huettig, editors, *Attention and Vision in Language Processing*, chapter 5, pages 77–96. Springer, New Delhi, 2015.

[23] R. Oostenveld, P. Fries, E. Maris, and J.-M. Schoffelen. FieldTrip: Open source software for advanced analysis of MEG, EEG, and invasive electrophysiological data. *Computational Intelligence and Neuroscience*, 2011(156869), 2011.

[24] M. J. Pickering and V. S. Ferreira. Structural priming: A critical review.

Psychological Bulletin, 134(3):427–459, 2008.

[25] M. Pokhoday, Y. Shtyrov, and A. Myachykov. Effects of visual priming and event orientation on word order choice in Russian sentence production. *Frontiers in Psychology*, 10(1661), 2019.

[26] R Core Team. *R: A language and environment for statistical computing*. R Foundation for Statistical Computing, Vienna, 2018.

[27] T. S. Redick, J. M. Broadway, M. E. Meier, P. S. Kuriakose, N. Unsworth, M. J. Kane, and R. W. Engle. Measuring working memory capacity with automated complex span tasks. *European Journal of Psychological Assessment*, 28(3):164–171, 2012.

[28] J. Sassenhagen and P. M. Alday. A common misapplication of statistical inference: Nuisance control with null-hypothesis significance tests. *Brain and Language*, 162:42–45, 2016.

[29] S. Sauppe. Word order and voice influence the timing of verb planning in German sentence production. *Frontiers in Psychology*, 8(1648), 2017.

[30] C. E. Shannon. A mathematical theory of communication. *The Bell System Technical Journal*, 27(3):379–423, 1948.

[31] M. van de Velde, A. S. Meyer, and A. E. Konopka. Message formulation and structural assembly: Describing "easy" and "hard" events with preferred and dispreferred syntactic structures. *Journal of Memory and Language*, 71(1):124–144, 2014.

[32] T. von der Malsburg. *saccades: Detection of Fixations in Eye-Tracking Data*, 2015. R package version 0.1-1.

[33] Z. Ye and X. Zhou. Executive control in language processing. *Neuroscience & Biobehavioral Reviews*, 33(8):1168–1177, 2009.
